# Calcific Uremic Arteriolopathy: A Case Series and Review from an Inner-City Tertiary University Center in End-Stage Renal Disease Patients on Renal Replacement Therapy

**DOI:** 10.1155/2021/6661042

**Published:** 2021-02-12

**Authors:** Mohamed Omer, Zeenat Yousuf Bhat, Nanette Fonte, Nashat Imran, James Sondheimer, Yahya Osman‐Malik

**Affiliations:** ^1^Division of Infectious Diseases, Mayo Clinic, Jacksonville, Florida, USA; ^2^Division of Nephrology and Hypertension, Wayne State University, Detroit Medical Center, Detroit, USA; ^3^Antelope Valley Nephrology Medical Group, Lancaster, California, USA

## Abstract

**Materials and Methods:**

24 patients with CUA and on RRT were evaluated at Detroit Medical Center from 2007 to 2016. Skin biopsy was used in almost all patients, along with the radiological and clinical findings. The patient's clinical and paraclinical data were retrieved from the electronic medical records. The effect of treatment modalities and the underlying hyperparathyroidism management were compared to the clinical outcomes using appropriate statistical tests.

**Results:**

Twenty-one patients were on hemodialysis, two patients received renal transplants, and one patient was on peritoneal dialysis. Diabetes mellitus was the most prevalent cause of ESRD. The parathyroid hormone level (PTH) was elevated in 22 patients. Fifteen patients were diagnosed 2 weeks or more prior to skin lesion onset. Twenty-two and thirteen patients received sodium thiosulphate and cinacalcet, respectively. Patients with lower PTH and the calcium-phosphate product levels had a relatively better outcome of CUA.

**Conclusions:**

*A m*ultifaceted approach may play a role in treating CUA. Referral to a nephrologist may aid in the early diagnosis and prompt management of CUA.

## 1. Introduction

Calcific uremic arteriolopathy, formerly called calciphylaxis, is an uncommon wrecking condition seen often in patients with advanced CKD, predominantly in patients on some form of renal replacement therapy (RRT) [1, 2]. It is initially characterized by livedo reticularis that advances to patches of ischemic necrosis, predominantly over the legs, thighs, gluteal region, abdomen, and breasts. It was first described in 1898 by Bryant and White [3]. The expression “calciphylaxis” was introduced by Selye in 1962 when he observed that inducing hyperparathyroidism or hypervitaminosis D in rodents could potentially lead to the progress of soft tissue calcification when exposed to injury or metallic salts, particularly iron therapy [4, 5]. The condition is also known as “metastatic calcinosis cutis” and “necrotizing or calcifying panniculitis.” Although it was thought to be uncommon in the past, the rate of CUA seems to rise, as proposed by an analysis of the United States Renal Data System [6]. A cross-sectional study of 242 patients undergoing hemodialysis in an outpatient setting presented a prevalence of 4 percent [7]. It was recently found that hypersensitivity or IgE release has no role in its development; subsequently, this eventually led to a significant change in the terminology to CUA, which was proposed as a better description of the underlying pathological process. A plethora of case reports published in the literature in the past addressed the same condition in patients without severe renal failure; a systematic review of 36 case reports discussed the development of nonuremic calciphylaxis [8]. CUA is characterized by regions of painful ischemic necrosis [9, 10]. As far as we can tell, in certain patients, pain may be the initial symptom before the development of the ulcerative lesions by numerous days. The exact mechanism of pain in CUA is a debatable issue and is believed to be ischemic in origin, yet, there might be a neuropathic element [11]. Skin lesions may have a particular preference for areas with abundant fat distribution. This has been shown in one of the most massive CUA reviews, including the leg, abdomen, and buttocks in 60, 23, and 9 percent of cases, respectively [12]. The variety of the lesions can be quite polymorphic and could be related to the stage of presentation. For instance, one of the most distinctive lesions is violaceous, painful, plaque-like subcutaneous nodules that may advance into frankly ischemic/necrotic ulcerative lesions with eschars once vascular thrombosis has been superimposed [13]. The risk of infection in eschars is relatively high [14]. Ischemic changes may appear early as livedo reticularis [15]. Less commonly, painful proximal muscle weakness can be the only presenting symptom shortly followed by skin lesions [16]. Both morbidity and mortality are quite unfavorable in this condition, with the most frequent cause of death being overwhelming sepsis because of compromised integrity of both epidermis and dermis [17].

Inducing hyperparathyroidism in animals was shown to increase the risk of ischemic skin necrosis [18]. Furthermore, parathyroidectomy has led to clinical improvement in many cases [1, 19]. Poorly controlled secondary hyperparathyroidism has been associated with significant complications like soft tissue and vascular calcification [20].

Many studies and case reports have linked warfarin as an essential risk factor for the precipitation of CUA. Iron administration has also been proposed as a risk factor for calciphylaxis. Iron deposits have been demonstrated in tissue samples from patients with calciphylaxis [21]. Moreover, excessive consumption of vitamin D was associated with an increased risk of subsequent development of CUA [2]. Skin biopsy is the gold standard with the other tools, including radiological scanning, which may show calcific radiopaque deposits at the same site of the skin lesions. Furthermore, clinical bedside diagnosis is remarkably helpful in the initial suspicion of this disorder.

Superimposed infections and sepsis are the leading cause of mortality [22]. Patients who survive severe ulcerative lesions might end up with significant scarring and disfigurement. Few cases might end up with extremity amputations at multiple levels.

The degree of skin involvement has been shown to directly proportionate with the one-year mortality rate that reaches up to 67% in deeper ulcerative lesions [23]. Earlier timing to the diagnosis and initiation of therapy has a significant impact on the final outcome, as well as the location of the lesions (proximal vs. distal), which has not been well examined.

## 2. Materials and Methods

The study was conducted in a tertiary inner-city university center, both retrospective as well as prospective over a 9-year period (2007–2016), and a total of 24 patients were enrolled in the study.

Following the Wayne State University/Detroit medical center's IRB approval, the ICD coding 9/10 systems were used to find patients admitted with CUA to the DMC hospitals, with no restriction on sample size or study duration. Inclusion criteria included age group of 18–80 years, patients with RRT for at least three months, and patients with a diagnosis of calcific uremic arteriolopathy. Exclusion criteria included loss of follow-up.

Demographic features of the patients including age, ethnicity, RRT modality, as well as the history of diabetes mellitus (DM), hypertension (HTN), peripheral artery disease (PAD), coronary artery disease (CAD), congestive heart failure (CHF), and cerebrovascular disease (CVA) were collected from their medical records.

The following variables were collected: serum calcium, phosphorus, calcium-phosphate product, and intact parathyroid hormone (iPTH). We also recorded serum ferritin, transferrin saturation%, protein C/S levels, and antiphospholipid antibody levels if available. Different renal osteodystrophy therapy methods were also recorded, including vitamin D therapy, oral calcimimetics, and subtotal parathyroidectomy. We examined the diagnosis methods of calciphylaxis, including clinical bedside evaluation, radiological imaging, and skin biopsy. Timing of the diagnosis, early (<2 weeks) vs. delayed (>2 weeks), and the anatomical site (proximal vs. distal) of the lesions were also noted. Components of multifaceted regimens that our patients received are sodium thiosulphate, hyperbaric O2, wound care, and/or modes of hyperthyroidism management, which were examined. Disease course and the final outcome of the lesions were categorized into complete resolution, partial resolution, or amputation and death. A critical guide to case series reporting was used to improve the quality of the study. We summarized the categorical variables using the number and percentage of the patients included in the study. Comparisons of the clinical characteristics of our patients were made using Fisher's exact test. All statistical tests were two-sided. The statistically significant *P* value was set to be less than 0.05. Our data were analyzed using SPSS version 27.

## 3. Results

As shown in Table 1, 24 patients were diagnosed with calcific uremic arteriolopathy (CUA). The results showed a female predominance of 18 patients as compared to 6 male patients. The mean age was 56.3 ± 14.6 years, ranging from 28 to 81. Ten patients were above 60 years of age, ten patients were aged between 40 and 60 years, and only four patients were less than 40 years. Among those cases, 22 were of African American descent, whereas only 2 cases were Caucasians. Twenty-one of our patients were on hemodialysis, only one patient managed through peritoneal dialysis, and the remaining 2 had a renal transplant. Diabetes mellitus (DM) was the most common etiology of end-stage renal disease (ESRD) with 13 cases being identified, while 8 cases had hypertension (HTN), with the three remaining reported etiologies as polycystic kidney disease (PKD), systemic lupus erythematosus (SLE), and focal segmental glomerulosclerosis. Seventeen patients had higher serum calcium of more than 8.5 mg/dl, and only seven patients had lower serum calcium of less than 8.5 mg/dl at the time of the diagnosis. Fifteen patients had lower serum phosphorus levels of less than 6.5 mg/dl, while nine patients had higher serum phosphorus levels of more than 6.5 mg/dl.

Moreover, 10 cases were diagnosed with a higher calcium-phosphate product of more than 55 mg^2^/dl^2^, and 14 patients had a lower calcium-phosphate product of less than 55 mg^2^/dl^2^. Most cases (*N* = 22 cases) had an elevated iPTH level compared to only 2 cases with normal iPTH levels. Nine patients had a serum PTH of more than 600 pg/ml, while 15 patients had a lower iPTH level of less than 600 pg/ml. Fourteen patients had a ferritin level of more than 500 *μ*g/l, while six patients had a ferritin level of less than 500 *μ*g/l. Fourteen patients had more than 20% transferrin saturation, while six patients had transferrin saturation less than 20%.

As Table 2 shows, 17 patients had histopathological evidence of CUA, while 4 cases were diagnosed based on bedside clinical examination, and only 3 cases were diagnosed based on radiological scanning. Regarding the time of diagnosis, 15 patients were diagnosed late (i.e., more than two weeks from the onset of the lesions), and nine patients were diagnosed earlier (i.e., less than two weeks from the onset of the lesions). From the anatomical standpoint, 13 patients had CUA lesions distal to the knee, whereas 11 patients had proximal lesions (above the knee).

As Table 3 shows, regarding the treatment of hyperparathyroidism, 13 patients were on cinacalcet, while only 3 received vitamin D analogs, alendronate, and parathyroidectomy in each arm, respectively. Eight out of our case series did not receive any mode of therapy for hyperparathyroidism. Only 2 cases received treatment with the HBO2. The majority of our case series were on sodium thiosulphate treatment (*n* = 22). 21 out of our cases received wound care management of their lesions. Regarding the outcome and progression of CUA lesions, 12 of our patients improved entirely or partially, while the other half of the cases ended up with amputation or died.

As shown in Table 4, patients with a lower PTH level of less than 600 pg/ml had an improvement in the outcome of their lesions (7/10: 70%). Nevertheless, patients who underwent amputation or died were more likely (7/12: 58.3%) to have PTH of less than 600 pg/ml. Of the two patients who showed partial improvement, one patient had a PTH level of more than 600 pg/ml, while the other had a PTH less than 600 pg/ml. Patients with a lower calcium-phosphate product (of less than 55 mg^2^/dl^2^) were found to have much more improvement (7/10: 70%), while patients who had calcium-phosphate products of more than 55 mg^2^/dl^2^ were less likely to survive or end up with more amputations (7/12: 58.3%). Only two patients with a lower calcium-phosphate product of less than 55 mg^2^/dl^2^ showed partial improvement. 8/10 (80%) of our patients with a lower phosphate level of less than 6.5 mg/dl were more likely to show improvement in the outcome, while 7/12 (58.3%) of our patients with a severely elevated phosphate level of more than 6.5 mg/dl were more likely to die or have an amputated limb. Only two patients showed partial improvement and had a phosphate level of less than 6.5 mg/dl. Significant differences were observed regarding the timing of the diagnosis and the final outcome of the lesions. Seven out of ten patients (70%) who had an improvement in the final outcome of their lesions were diagnosed in less than 2 weeks. Ten out of twelve patients (83.3%) who had a poor outcome, i.e., had amputated limb or expired, had a late diagnosis in their course. *P* value = 0.019.

As illustrated in Table 5, notably, patients who did not receive any parathyroid treatment were more likely (5/8: 62.5%) to have a lower phosphate level of less than 6.5 mg/dl. In comparison, patients who received cinacalcet as a form of therapy were more likely (8/13: 62%) to have a lower phosphate level of less than 6.5 mg/dl. Patients who were on Doxercalciferol and Alendronate were found to have a lower phosphate level of less than 6.5 mg/dl as well. One patient underwent parathyroidectomy and had a phosphate level of more than 6.5 mg/dl. Patients who did not receive any form of parathyroid therapy were more likely (7/8 : 85.5%) to have a lower PTH level of less than 600 pg/ml, as well as patients who were on cinacalcet therapy, who also more likely (7/13: 53.8%) had a PTH level of less than 600 pg/ml. Only one patient received Doxercalciferol and had severely elevated PTH levels of more than 600 pg/ml. One patient underwent parathyroid gland resection and had an elevated PTH level of more than 600 pg/ml as well. Finally, only one patient received Alendronate and had a PTH level of less than 600 pg/ml.

As Table 6 shows, the use of multifaceted treatment and its relation to the outcome, 14 patients received three different regimens for the management of CUA, while 9 received two regimens, and one patient received wound care only as a mode of management.

## 4. Discussion

CUA is a syndrome characterized by both thrombosis and small vessel calcification that significantly burdens patients with end-stage renal disease resulting in high morbidity and mortality. It is well known to be multifactorial in etiology. Our findings from this study suggest that many risk factors may predispose patients to the development of CUA. A significant number of patients have shown improvement with the multidisciplinary approach of those lesions. Our study has shown that most of the patients affected were of the female sex, which is in line with numerous previous studies in the past [14, 24]. The fat distribution pattern might explain the prevalence of CUA lesions in females, particularly those with an average BMI of more than 30 [3]. Our study has demonstrated that most of our patients were of African American descent compared to other studies in which patients were predominantly Caucasians [13, 25–27]. However, our tertiary hospital demographically deals with patients who are predominantly of African American origin, which may explain those findings. Most of our patients were aged above 55, which is similar to other studies in the literature that suggested advanced age as a contributing factor [25]. Most of our patients had multiple comorbidities. As reported in previous studies, diabetes mellitus was the commonest contributing factor that may play a role in the development and progression of the lesions making management quite difficult in these subtypes of patients [2, 3, 24]. Early skin lesions like subcutaneous nodules in patients with end-stage renal disease, secondary hyperparathyroidism, calcium, and phosphorus abnormalities should raise the clinicians' suspicion about the possibility of CUA diagnosis. CUA lesions can mimic a wide variety of skin pathologies, including atherosclerosis, cholesterol embolization, warfarin skin necrosis, endarteritis obliterans, vasculitis, cellulitis, purpura fulminans, oxalate vasculopathy, antiphospholipid antibody syndrome, radiation arteritis, Martorell hypertensive ischemic ulcer, cardiac myxoma, and in early nephrogenic systemic fibrosis skin lesions [11, 28].

Ghosh et al. [29] have shown that CUA may be misdiagnosed in its early stages. An appropriate skin biopsy remains the gold standard to confirm a CUA diagnosis to distinguish it from other conditions. 17 of our patients were diagnosed by skin biopsy, and only three were identified via radiological imaging based on the patterns of calcium deposits in the site of CUA lesions. However, the diagnostic utility is not established yet [30, 31], while 4 cases were diagnosed based on bedside clinical examination only.

Most of the patients in this cohort were treated with hemodialysis, which suggests more incidence of CUA with this modality of renal replacement therapy. However, one single-center study had shown a higher incidence in patients on peritoneal dialysis [32]. The high incidence of CUA lesions may be explained by a heavier calcium exposure in this subset of patients.

In our study, we identified only seven patients who were hypercalcemic at the time of diagnosis; this is in contrast to a study published by Nigwekar et al. [26], which had found that hypercalcemia was common in most of their patients with an OR of 1.83 (*P*: 0.02).

Most of our patients' lab values were within the normal range at the time of diagnosis, and this is a major shortcoming in accurately addressing the immediate risk factor to predispose patients to the development of CUA lesions. Although many studies had implicated the role of hyperphosphatemia in the development of CUA (3) [33], 15 of our patients had serum phosphorous levels of less than 6.5 mg/dl.

In our case series, almost all of our patients (*n* = 22) had significant hyperparathyroidism, which is in keeping with many previous studies showing its major role in the development of CUA lesions (3) [33]. We believed that moderate to severe hyperparathyroidism predispose renal failure patients to the development of CUA lesions. A high parathyroid hormone level induces high-turnover bone disease, which is significantly associated with vascular calcification in renal failure patients and eventually may lead to the development of CUA lesions; however, a low level (<100 pg/ml) induces adynamic bone disease, which is less associated with the development of CUA lesions. Abnormalities in calcium, phosphorus, or PTH may be noted in patients with calciphylaxis, but it is not always the case, as these patients may present with normal lab values. In our study, the most notable abnormality is the elevated parathyroid hormone levels.

This study found that the majority of our patients with improved outcomes had PTH levels of less than 600 pg/ml; nevertheless, when considering patients who expired or had amputations with similar PTH levels, this observation was statistically insignificant. *P* value = 0.79.

The majority of our patients (7/10: 70%) who had an improvement in the final outcome of their lesions were diagnosed in less than 2 weeks. Most of the patients (10/12: 83.3%) who had a poor outcome, i.e., had amputated limb or expired, had a late diagnosis in their course. This relationship showed statistical significance. *P* = 0.019.

Many reports had addressed the beneficial role of reducing phosphate and calcium-phosphate product in the outcome of CUA lesions [34–36]; this is well shown in our study, which has found better outcomes in patients with a lower calcium-phosphorus product of less than 55 mg^2^/dl^2^. Block et al. have shown that patients with a calcium-phosphate product of higher than 72 mg^2^/dl^2^ had a relatively higher risk of mortality [37].

A paper published by Farah et al. [38] has found, in 12 histological tissue samples of CUA lesions, significant iron deposits affecting the microvasculature but otherwise absent in other tissues. This finding raises the hypothesis that iron-inducing oxidative stress in patients with uremia and iron-chelating therapy might play a role in the treatment of CUA.

Zarjou et al. [39] concluded that ferritin might help to prevent vascular calcification by the induction of a heme-oxygenase-1/ferritin system that prevents inorganic phosphate mediated calcification and osteoblastic differentiation of human smooth muscle cells mainly via ferroxidase activity of ferritin. In our study, 14 of our patients had elevated ferritin of more than 500 *μ*g/l and transferrin saturation of >20%.

We cannot overemphasize the importance of a high index of CUA suspicion in patients with deranged bone and mineral profiles on renal replacement therapy. This subset of the patient needs an urgent referral to a nephrologist to reach a final diagnosis. Out of 24 patients, only 9 cases (37.5%) were diagnosed earlier than two weeks with CUA from the development of the lesions, which is in keeping with the same results by Ghosh et al. [29], who identified only two out of five patients early in their course of the development of the lesions, and hence receiving prompt treatment is associated with less grievous complications.

With the use of multifaceted interventions, 10 of our patients showed complete recovery from CUA lesions, while two patients have made a partial recovery. However, 12 patients have taken a relatively steep downhill course with amputation of an extremity as well as death. This particular subset could have a heavier comorbid load. Unfortunately, it is difficult to track the exact cause of their death due to a lack of information in our medical record. From our case series, there was no specific combination of therapies that pointed toward the improvement of CUA lesions.

A literature review on 104 cases was conducted and showed that parathyroidectomy remains the most important modality to improve patients with severe hyperparathyroidism and CUA, as suggested by Hafner et al. [40]. The vast majority of our patients did not receive parathyroidectomy as a form of parathyroid treatment (only one patient did), and this might be explained by the availability of more potent medical therapy such as cinacalcet. Patients who received cinacalcet were more likely to have PTH of less than 600 pg/ml. Higgins et al. pointed out that the recurrence of severe hyperparathyroidism is less likely to occur following parathyroid surgery [41].

To our knowledge, no study significantly addressed in detail a therapy for CUA. The management of such a condition depends mainly on the multifaceted interventions to treat CUA lesions as suggested by many other studies, leading to favorable outcomes [42–44]. Thirteen of our patients received cinacalcet as a treatment for hyperparathyroidism and CUA. Several case reports showed the benefits of cinacalcet [45–47]; however, a meta-analysis for the use of cinacalcet in the management of patients with CUA has shown no association with a lower risk of mortality, wound progressions, and amputation [48]. Sodium thiosulphate is an inorganic salt with significant antioxidant properties and a vasodilatory effect [49]. Twenty-two of our patients received STS in addition to the other therapy modes, with 12 patients showing a significant response. Hyperbaric oxygen therapy involves administering 100% oxygen in a pressurized environment (hyperbaric chamber) (>1.4 absolute atmosphere ATA). In this way, oxygen transport is increased 27 times compared with the O_2_ transport in plasma. This type of therapy favors the healing of CUA lesions with remarkable improvement in pain control. In addition, HBO_2_ therapy increases host and cellular immune responses and fibroblast-mediated collagen production and enhances the action of antibiotic therapy. In our study, only two patients could be enrolled in this therapy type due to our institution's logistic restrictions. The two patients showed a significant improvement in wound healing and less pain, which is in keeping with few studies published in the past [50–52]. A meta-analysis examining a few cohorts has failed to show its risk-reducing effect in the progression of skin lesions, amputation, or death [48].

## 5. Conclusion

CUA is associated with a high rate of morbidity and mortality in patients with end-stage renal disease. The lesions associated with this disorder are usually misdiagnosed by the clinicians, which leads to a significant delay in therapy initiation. Early diagnosis and referral to a nephrologist will guarantee to deliver prompt treatment and prevent lethal complications. Overall, the importance of good control of mineral and bone profile is a cornerstone of preventing CUA. Based on our results, we strongly recommend a multidisciplinary approach to both diagnosis and therapy of CUA, which can lead to a less unfavorable outcome. CUA multifaceted therapy may play a role in the final outcome of the lesions, and clearly, from our case series, no single patient has received all the modalities of CUA therapy. It must be borne in mind that this study was only conducted in a small group of patients. More case-control multicenter clinical trials are required to unravel numerous mysterious issues regarding CUA.

## Figures and Tables

**Table 1:** Baseline characteristics and biochemical lab values: (*n* = 24) unless noted.

Patient variables	Frequency (%)
Gender	
Male	6 (25%)
Female	18 (75%)

Age group (years)	
<40	4 (16.7%)
40–60	10 (41.7%)
>60	10 (41.7%)

Ethnicity	
African american	22 (91.7%)
Caucasian	2 (2.3%)

Mode of renal replacement therapy	
Hemodialysis	21 (87.5%)
Peritoneal dialysis	1 (4.2%)
Renal transplant	2 (8.3%)

ESRD etiology	
Diabetes	13 (54.2%)
Hypertension	8 (33.3%)
Others	3 (12.3%)

Calcium level (mg/dl)	
Ca > 8.5	17 (70.8%)
Ca < 8.5	7 (29.2%)

Phosphate level (mg/dl)	
Ph > 6.5	9(37.5%)
Ph < 6.5	15(62.5%)

Calcium-phosphate product level (mg^2^/dl^2^)	
CaPP > 55	10 (41.7%)
CaPP < 55	14 (58.3%)

Parathyroid hormone level (pg/ml)	
PTH > 600	9 (37.5%)
PTH < 600	15 (62.5%)

Ferritin level (*μ*g/l) (*n* = 20)^*∗*^	
Ferritin > 500	14 (58.3%)
Ferritin < 500	6 (25%)

Transferrin saturation (%) (*n* = 20)^*∗*^	
T.sat > 20%	14 (58.3%)
T.sat < 20%	6 (25%)

Ca: serum calcium, Ph: serum phosphorus, CaPP: serum calcium-phosphorus products, PTH: serum parathyroid hormone, T.Sat: transferrin saturation. ^*∗*^4 cases were missed to follow-up of transferrin saturation and ferritin levels.

**Table 2:** The mode, the timing of the diagnosis, and site of the lesions.

Mode of the diagnosis	
Skin biopsy	17 (70.8%)
Imaging	3 (12.5%)
Clinical	4 (16.7%)
Timing of the diagnosis	
Early (<2 weeks)	9 (37.5%)
Late (>2 weeks)	15 (62.5%)
Site of the lesion	
Proximal (above the knee)	11 (45.8%)
Distal (below the knee)	13 (54.2%)

**Table 3:** Types of therapy to manage hyperparathyroidism and the outcome of the CUA lesions.

Patient variables		Frequency (%)
Therapy of hyperparathyroidism		
Vitamin D		1 (4.2%)
Cinacalcet		13 (54.2%)
Parathyroidectomy		1 (4.2%)
Alendronate		1 (4.2%)
No treatment		8 (33.3%)

Therapy of calcific uremic arteriolopathy^*∗*^		
	Received	Did not receive
HBO_2_	2 (8.3%)	22 (91.7%)
Cinacalcet	13 (54.1%)	11 (45.9%)
Wound care	21 (87.5%)	3 (12.5%)
NaTSO_4_	22 (91.7%)	2 (8.3%)
PTHx	1 (4.2%)	23 (95.8%)

The outcome of the lesion		
Improved		10 (41.7%)
Partially improved		2 (8.3%)
Expired or amputation		12 (50%)

HBO: hyperbaric oxygen therapy, NaTSO_4_: sodium thiosulphate, PTHx: parathyroidectomy. ^*∗*^Patients may be enrolled in multiple types of treatment for calcific uremic arteriolopathy.

**Table 4:** The outcome of calcific uremic arteriolopathy lesions in relation to the severity of hyperparathyroidism, levels of calcium-phosphate products, the degree of hyperphosphatemia, and the timing of the diagnosis.

Outcome	Parathyroid hormone levels				*P* value
		<600	>/=600	Total	
	Improved	7 (70%)	3 (30%)	10 (100%)	0.79
	Partial improvement	1 (50.00%)	1 (50.00%)	2 (100%)	
	Expired or amputation	7 (58.3%)	5 (41.7%)	12 (100%)	
Total		15	9	24	

Outcome	Calcium-phosphate product levels				
		<55	>/=55	Total	
	Improved	7 (70%)	3 (30%)	10 (100%)	0.18
	Partial improvement	2 (100%)	0 (0.00%)	2 (100%)	
	Expired/Amputated	5 (41.7%)	7 (58.3%)	12 (100%)	
Total		14	10	24	

Outcome	Phosphate levels				
		<6.5	>6.5	Total	
	Improved	8 (80%)	2 (20%)	10 (100%)	0.94
	Partial improvement	2 (100%)	0 (0.00%)	2 (100%)	
	Expired/Amputated	5 (41.7%)	7 (58.3%)	12 (100%)	
Total		15	9	24 (100%)	

Outcome	Timing of diagnosis				
		Early	Late	Total	
	Improved	7 (70%)	3 (30%)	10 (100%)	0.019
	Partial improvement	0 (0.00%)	2 (100%)	2 (100%)	
	Expired/Amputated	2 (16.7%)	10 (83.3%)	12(100%)	
Total		9 (37.5%)	15 (62.5%)	24 (100%)	

**Table 5:** The relation of parathyroid treatment to the degree of hyperphosphatemia and parathyroid hormone levels.

Parathyroid treatment	Phosphate levels				*P* value
		<6.5	>/= 6.5	Total	

	No treatment	5(62.5%)	3(37.5%)	8(100%)	0.58
	Cinacalcet	8(62%)	5(38%)	13(100%)	
	Doxercalciferol	1(100%)	0(0.00%)	1(100%)	
	Parathyroidectomy	0(0.00%)	1(100%)	1(100%)	
	Alendronate	1(100%)	0(0.00%)	1(100%)	
Total		15	9	24	

Parathyroid treatment	Parathyroid hormone levels				
		<600	>/= 600	Total	
	No treatment	7(85.5%)	1(12.5%)	8(100%)	0.16
	Cinacalcet	7(53.8%)	6(46.2%)	13(100%)	
	Doxercalciferol	0(0.00%)	1(100%)	1(100%)	
	Parathyroidectomy	0(0.00%)	1(100%)	1(100%)	
	Alendronate	1(100%)	0(0.00%)	1(100%)	
Total		15	9	24	

**Table 6:** The relationship of multifaceted regimens of our patients and their final outcomes.

Patients	Sodium thiosulfate	Hyperbaric oxygen	Wound care	Hyperparathyroidism management	Outcome
Patient no. 1	√	—	√	Cinacalcet	Improved
Patient no. 2	√	—	√	Cinacalcet	Improved
Patient no. 3	√	—	√	Cinacalcet	Amputated/Died
Patient no. 4	√	—	—	Cinacalcet	Partially improved
Patient no. 5	√	—	√	—	Amputated/Died
Patient no. 6	√	—	—	Cinacalcet	Amputated/Died
Patient no. 7	√	—	√	—	Amputated/Died
Patient no. 8	√	—	√	Cinacalcet	Amputated/Died
Patient no. 9	√	—	√	Cinacalcet	Amputated/Died
Patient no. 10	√	—	√	Doxercalciferol	Improved
Patient no. 11	√	√	√	—	Amputated/Died
Patient no. 12	√	—	√	—	Improved
Patient no. 13	√	—	√	—	Amputated/Died
Patient no. 14	√	—	√	Parathyroidectomy	Amputated/Died
Patient no. 15	√	—	√	Cinacalcet	Amputated/Died
Patient no. 16	√	—	√	Cinacalcet	Partially improved
Patient no. 17	√	—	—	Cinacalcet	Improved
Patient no. 18	√	—	√	Cinacalcet	Improved
Patient no. 19	√	—	√	Alendronate	Amputated/Died
Patient no. 20	—	—	√	—	Improved
Patient no. 21	√	—	√	Cinacalcet	Improved
Patient no. 22	√	—	√	Cinacalcet	Amputated/Died
Patient no. 23	√	—	√	—	Improved
Patient no. 24	—	√	√	—	Improved

## Data Availability

The data used to support this article will be provided upon reasonable request from the corresponding author.

## References

[1] McCarthy J. T., Patzelt M. T., el-Azhary R. A., Weaver A. L. (2016). Survival, risk factors, and effect of treatment in 101 patients with calciphylaxis. *Mayo Clinic Proceedings*.

[2] Fine A., Zacharias J. (2002). Calciphylaxis is usually non-ulcerating: risk factors, outcome and therapy. *Kidney International*.

[3] Bryant J. H., White W. H. (1899). A case of calcification of the arteries and obliterative endarteritis, associated with hydronephrosis, in a child aged six months. *Guys Hospital*.

[4] Selye H., Gabbiani G., Strebel R. (1962). Sensitization to calciphylaxis by endogenous parathyroid hormone. *Endocrinology*.

[5] Selye H. (1962). The dermatologic implications of stress and calciphylaxis. *Journal of Investigative Dermatology*.

[6] Nigwekar S. U., Solid C. A., Ankers E. (2014). Quantifying a rare disease in administrative data: the example of calciphylaxis. *Journal of General Internal Medicine*.

[7] Angelis M., Wong L. L., Myers S. A., Wong L. M. (1997). Calciphylaxis in patients on hemodialysis: a prevalence study. *Surgery*.

[8] Nigwekar S. U., Wolf M., Sterns R. H., Hix J. K. (2008). Calciphylaxis from nonuremic causes: a systematic review. *Clinical Journal of the American Society of Nephrology*.

[9] Jeong H. S., Dominguez A. R. (2016). Calciphylaxis: controversies in pathogenesis, diagnosis and treatment. *The American Journal of the Medical Sciences*.

[10] Janigan D. T., Hirsch D. J., Klassen G. A., MacDonald A. S. (2000). Calcified subcutaneous arterioles with infarcts of the subcutis and skin (“calciphylaxis”) in chronic renal failure. *American Journal of Kidney Diseases*.

[11] Nigwekar S. U., Kroshinsky D., Nazarian R. M. (2015). Calciphylaxis: risk factors, diagnosis, and treatment. *American Journal of Kidney Diseases*.

[12] Galloway P. A. G., El-Damanawi R., Bardsley V. (2015). Vitamin K antagonists predispose to calciphylaxis in patients with end-stage renal disease. *Nephron*.

[13] Ahmed S., O’Neill K. D., Hood A. F., Evan A. P., Moe S. M. (2001). Calciphylaxis is associated with hyperphosphatemia and increased osteopontin expression by vascular smooth muscle cells. *American Journal of Kidney Diseases*.

[14] Nigwekar S. U., Brunelli S. M., Meade D., Wang W., Hymes J., Lacson E. (2013). Sodium thiosulfate therapy for calcific uremic arteriolopathy. *Clinical Journal of the American Society of Nephrology*.

[15] Wilmer W. A., Magro C. M. (2002). Calciphylaxis: emerging concepts in prevention, diagnosis, and treatment. *Seminars in Dialysis*.

[16] Edelstein C. L., Wickham M. K., Kirby P. A. (1992). Systemic calciphylaxis presenting as a painful, proximal myopathy. *Postgraduate Medical Journal*.

[17] Oh D. H., Eulau D., Tokugawa D. A., McGuire J. S., Kohler S. (1999). Five cases of calciphylaxis and a review of the literature. *Journal of the American Academy of Dermatology*.

[18] Selye H., Somogyi A., Mecs I. (1968). Calcergy inhibited by calciphylactic challengers. *Science*.

[19] Arch-Ferrer J. E., Beenken S. W., Rue L. W., Bland K. I., Diethelm A. G. (2003). Therapy for calciphylaxis: an outcome analysis. *Surgery*.

[20] Goodman W. G. (2004). The consequences of uncontrolled secondary hyperparathyroidism and its treatment in chronic kidney disease. *Seminars in Dialysis*.

[21] Amuluru L., High W., Hiatt K. M. (2009). Metal deposition in calcific uremic arteriolopathy. *Journal of the American Academy of Dermatology*.

[22] Adrogué H. J., Frazier M. R., Zeluff B., Suki W. N. (1981). Systemic calciphylaxis revisited. *American Journal of Nephrology*.

[23] Smith J., Findlay M., Geddes C., Fox J. (2012). The role of sodium thiosulphate in the treatment of calciphylaxis. *Portuguese Journal of Nephrology & Hypertension*.

[24] Floege J., Kubo Y., Floege A., Chertow G. M., Parfrey P. S. (2015). The effect of cinacalcet on calcific uremic arteriolopathy events in patients receiving hemodialysis: the EVOLVE trial. *Clinical Journal of the American Society of Nephrology*.

[25] Weenig R. H., Sewell L. D., Davis M. D. P., McCarthy J. T., Pittelkow M. R. (2007). Calciphylaxis: natural history, risk factor analysis, and outcome. *Journal of the American Academy of Dermatology*.

[26] Nigwekar S. U., Bhan I., Turchin A. (2013). Statin use and calcific uremic arteriolopathy: a matched case-control study. *American Journal of Nephrology*.

[27] Bleyer A., Choi M., Igwemezie B., de la Torre E., White W. (1998). A case control study of proximal calciphylaxis. *American Journal of Kidney Diseases*.

[28] Hafner J. (2016). Calciphylaxis and Martorell hypertensive ischemic leg ulcer: same pattern—one pathophysiology. *Dermatology*.

[29] Ghosh T., Winchester D. S., Davis M. D. P., El-Azhary R., Comfere N. I. (2017). Early clinical presentations and progression of calciphylaxis. *International Journal of Dermatology*.

[30] Bleibel W., Hazar B., Herman R. (2006). A case report comparing various radiological tests in the diagnosis of calcific uremic arteriolopathy. *American Journal of Kidney Diseases*.

[31] Halasz C. L., Munger D. P., Frimmer H., Dicorato M., Wainwright S. (2017). Calciphylaxis: comparison of radiologic imaging and histopathology. *Journal of the American Academy of Dermatology*.

[32] Zhang Y., Corapi K. M., Luongo M., Thadhani R., Nigwekar S. U. (2016). Calciphylaxis in peritoneal dialysis patients: a single center cohort study. *International Journal of Nephrology and Renovascular Disease*.

[33] Mazhar A. R., Johnson R. J., Gillen D. (2001). Risk factors and mortality associated with calciphylaxis in end-stage renal disease. *Kidney International*.

[34] Bleyer A. J., White W. L., Choi M. J. (2000). Calcific small vessel ischemic disease (calciphylaxis) in dialysis patients. *The International Journal of Artificial Organs*.

[35] Russell R., Brookshire M. A., Zekonis M., Moe S. M. (2002). Distal calcific uremic arteriolopathy in a hemodialysis patient responds to lowering of Ca x P product and aggressive wound care. *Clinical Nephrology*.

[36] Don B. R., Chin A. I. (2003). A strategy for the treatment of calcific uremic arteriolopathy (calciphylaxis) employing a combination of therapies. *Clinical Nephrology*.

[37] Block G., Hulbert-Shearon T., Levin N., Port F. (1998). Association of serum phosphorus and calcium x phosphate product with mortality risk in chronic hemodialysis patients: a national study. *American Journal of Kidney Diseases*.

[38] Farah M., Crawford R. I., Levin A., Chan Yan C. (2011). Calciphylaxis in the current era: emerging “ironic” features?. *Nephrology Dialysis Transplantation*.

[39] Zarjou A., Jeney V., Arosio P. (2009). Ferritin prevents calcification and osteoblastic differentiation of vascular smooth muscle cells. *Journal of the American Society of Nephrology*.

[40] Hafner J., Keusch G., Wahl C. (1995). Uremic small-artery disease with medial calcification and intimal hyperplasia (so-called calciphylaxis): a complication of chronic renal failure and benefit from parathyroidectomy. *Journal of the American Academy of Dermatology*.

[41] Shen W., Düren M., Morita E. (1996). Reoperation for persistent or recurrent primary hyperparathyroidism. *Archives of Surgery (Chicago, Ill: 1960)*.

[42] Russo D., Capuano A., Cozzolino M. (2016). Multimodal treatment of calcific uraemic arteriolopathy (calciphylaxis): a case series. *Clinical Kidney Journal*.

[43] Harris C., Kiaii M., Lau W., Farah M. (2018). Multi-intervention management of calcific uremic arteriolopathy in 24 patients. *Clinical Kidney Journal*.

[44] Baldwin C., Farah M., Leung M. (2011). Multi-intervention management of calciphylaxis: a report of 7 cases. *American Journal of Kidney Diseases*.

[45] Velasco N., MacGregor M. S., Innes A., MacKay I. G. (2006). Successful treatment of calciphylaxis with cinacalcet-an alternative to parathyroidectomy?. *Nephrology Dialysis Transplantation*.

[46] Robinson M. R., Augustine J. J., Korman N. J. (2007). Cinacalcet for the treatment of calciphylaxis. *Archives of Dermatology*.

[47] Sharma A., Burkitt-Wright E., Rustom R. (2006). Cinacalcet as an adjunct in the successful treatment of calciphylaxis. *British Journal of Dermatology*.

[48] Udomkarnjananun S., Kongnatthasate K., Praditpornsilpa K., Eiam-Ong S., Jaber B. L., Susantitaphong P. (2019). Treatment of calciphylaxis in CKD: a systematic review and meta-analysis. *Kidney International Reports*.

[49] Sowers K. M., Hayden M. R. (2010). Calcific uremic arteriolopathy: pathophysiology, reactive oxygen species and therapeutic approaches. *Oxidative Medicine and Cellular Longevity*.

[50] An J., Devaney B., Ooi K. Y., Ford S., Frawley G., Menahem S. (2015). Hyperbaric oxygen in the treatment of calciphylaxis: a case series and literature review. *Nephrology*.

[51] Basile C., Montanaro A., Masi M., Pati G., De Maio P., Gismondi A. (2002). Hyperbaric oxygen therapy for calcific uremic arteriolopathy: a case series. *Journal of Nephrology*.

[52] Podymow T., Wherrett C., Burns K. D. (2001). Hyperbaric oxygen in the treatment of calciphylaxis: a case series. *Nephrology Dialysis Transplantation*.

